# A Fanciful Juxtaposition, a Reimagined Farm

**DOI:** 10.3201/eid2512.AC2512

**Published:** 2019-12

**Authors:** Byron Breedlove

**Affiliations:** Centers for Disease Control and Prevention, Atlanta, Georgia, USA

**Keywords:** art science connection, emerging infectious diseases, art and medicine, about the cover, Joan Miró, The Tilled Field, a fanciful juxtaposition, a reimagined farm, novel pathogens, viruses, emerging and reemerging zoonotic infections, bacteria, parasites, fungi, anthrax, brucellosis, cryptosporidiosis, hantavirus pulmonary syndrome, leptospirosis, rabies, salmonellosis, zoonoses

**Figure:**
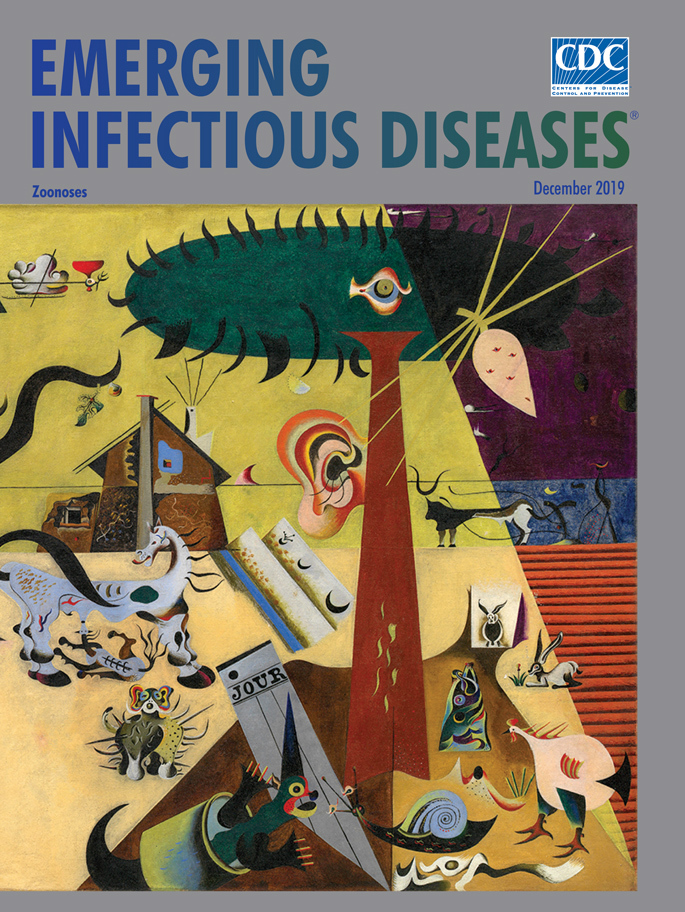
**Joan Miró (1893–1983), *The Tilled Field *(La Terre Labourée), 1923–1924.** Oil on canvas; 26 in × 36.5 in/66 cm × 92.7 cm. © 2018 Successió Miró / Artists Rights Society (ARS), New York / ADAGP, Paris 2019. Photo Credit: The Solomon R. Guggenheim Museum/Art Resource, New York, New York, USA.

Detecting the emergence of novel pathogens before they spread from local sites, where they first appear in animals or humans, is crucial for responding effectively to zoonotic health threats. In *The Tilled Field, *which appears as this month’s cover art, internationally acclaimed artist Joan Miró Ferra was not, of course, offering a lesson in how zoonoses such as anthrax, brucellosis, cryptosporidiosis, hantavirus pulmonary syndrome, leptospirosis, orf, rabies, or salmonellosis may be spread. Nonetheless, his painting reminds viewers of the interdependent relationship among people, animals, plants, and their shared environment. 

The Fundació Joan Miró notes that Miró “avoided academicism in his constant quest for a pure, global art that could not be classified under any specific movement.” Throughout his long career, Miró’s deliberate approach to, and tenacious experimentation with, new forms of expression enabled him to complete a vast, diverse collection of works, estimated to include some 2,000 oil paintings, 500 sculptures, 400 ceramic objects, 5,000 drawings and collages, and 250 illustrated books. Miró said, “I work like a labourer on a farm or in a vineyard. Things come to me slowly. My vocabulary of forms, for instance, has not been the discovery of a day. It took shape in spite of myself.... That is why I am always working on a hundred different things at the same time.” 

During the summer of 1923, Miró started painting *The Tilled Field*, an homage to his family’s farm in Mont-roig del Camp, Catalonia, Spain. Miró had previously approached the same subject in an earlier painting called *The Farm* (1921–1922). (Writer Ernest Hemingway, who purchased *The Farm*, wrote that “After Miró had painted *The Farm* and after James Joyce had written *Ulysses,* they had a right to expect people to trust the further things they did even when the people did not understand them.”) 

*The Tilled Field* is noteworthy both for being among Miró’s earliest surrealistic works and for marking his nascent use of an evolving pictorial language of symbols and creatures he employed throughout the rest of his career. Nancy Spector, chief curator and art director at the Guggenheim Museum, notes that the “fanciful juxtaposition of human, animal, and vegetal forms and its array of schematized creatures constitute a realm visible only to the mind’s eye and reveal the great range of Miró’s imagination.” 

Miró organized the painting into distinct areas defined by geometric shapes. Subdued, smooth trapezoid panels of dark and pale yellow converge at the center and fill most of the canvas, functioning as sky and earth, respectively. Six rippled furrows in the bottom left represent one tilled field. The crisp diagonal line that slices down the right side creates a triangle, subdivided into three distinct sections: another tilled field in the bottom right, a small blue trapezoid of blue sky (daylight) in the center, and a larger purple trapezoid (night) situated in the top right.

The large tree dominating the right side of the painting features an all-seeing eye centered in its biomorphic crown and a human ear attached to its trunk. The French word *jour* (day) appears on folded sheet of newsprint at the base of the tree; a farmhouse with cracked walls and a straight chimney―perhaps Miro’s family home―occupies the center of the canvas. To the left, a stylized tree cradles a flagpole with the flags of France, Spain, and Catalonia emerging from its crook. Another flag hangs between the tree limb that juts to the upper left of the canvas and the stalk thrusting up from the sawtooth aloe plant.

A menagerie of multicolored creatures, including a dog, snail, horse and foal, chicken, rabbits, birds, a fish half out of the water, and a lizard, are scattered across the painting. Many were inspired by various Catalan ceramic that Miró collected. Historical and cultural sources were also important to the artist, and the farmer following a cattle-drawn plough is styled on the Altamira cave paintings. Though Miró employs a surrealistic perspective, the overall impression is that his painting still evokes normal life on a family farm before the Spanish Civil War. Art historian Janis Mink notes that “the animals, house, fields, and plants have become disquieting presences, stretched, swollen, and barbed sometimes even into ugliness. At the same time, they insist on their identities.” 

Miró’s reimagined, surreal farm depicts a setting in which humans and animals would be in close proximity. It is the type of environment where emerging and reemerging zoonotic infections could be spread from between animals and humans via viruses, bacteria, parasites, or fungi. Zoonotic diseases are spread in myriad ways, from direct contact with animals or their blood, birth products, urine, or feces; being bitten or scratched by animals; encountering water or soil contaminated with pathogens spread by animals; or consuming unsafe or contaminated foods. All are possibilities on a small family farm or scaled-up modern agricultural enterprises.

## References

[1] Bird BH, Mazet JAK. Detection of emerging zoonotic pathogens: an integrated One Health approach. Annu Rev Anim Biosci. 2018;6:121–39. 10.1146/annurev-animal-030117-014628 29144769

[2] Centers for Disease Control and Prevention. Zoonotic diseases [cited 2019 Oct 22]. https://www.cdc.gov/onehealth/basics/zoonotic-diseases.html

[3] Centers for Disease Control and Prevention. One Health [cited 2019 Oct 26]. https://www.cdc.gov/onehealth/index.html

[4] Fundació Joan Miró. Biography [cited 2019 Oct 23]. https://www.fmirobcn.org/en/joan-miro/

[5] Guggenheim Museum. Joan Miró. The tilled field (*La terre labourée*) [cited 2019 Aug 23]. https://www.guggenheim.org/artwork/2934

[6] Hemingway E. The Farm. Paris. Cahiers d’Art. 1934;IX:28–9.

[7] Mink J. Joan Miró: 1893–1983. Cologne (Germany): Taschen; 2000. p. 37–9.

[8] Rattray J. A delicious imaginary journal with Joan Miró and Jose Maria Hinjosa. In: Havard R, editor. Companion to Spanish surrealism. Rochester (NY): Tamesis Books; 2004. p. 33–4.

[9] World Health Organization. Managing public health risks at the human-animal-environment interface [cited 2019 Oct 27]. https://www.who.int/zoonoses

